# Objective Video-Based Assessment of ADHD-Like Canine Behavior Using Machine Learning

**DOI:** 10.3390/ani11102806

**Published:** 2021-09-26

**Authors:** Asaf Fux, Anna Zamansky, Stephane Bleuer-Elsner, Dirk van der Linden, Aleksandr Sinitca, Sergey Romanov, Dmitrii Kaplun

**Affiliations:** 1 Information Systems Department, University of Haifa, Haifa 3498838, Israel; asaffoox@gmail.com (A.F.); vetbehavior.il@gmail.com (S.B.-E.); 2 Department of Computer and Information Sciences, Northumbria University, Newcastle upon Tyne NE7 7XA, UK; dirk.vanderlinden@northumbria.ac.il; 3 Department of Automation and Control Processes, Saint Petersburg Electrotechnical University “LETI”, 197376 Saint Petersburg, Russia; amsinitca@etu.ru (A.S.); saromanov@etu.ru (S.R.); dikaplun@etu.ru (D.K.)

**Keywords:** behavioral assessment, veterinary science, machine learning, ADHD-like behavior

## Abstract

**Simple Summary:**

This paper applies machine learning techniques to propose an objective video-based method for assessing the degree of canine ADHD-like behavior in veterinary consultation room. The method is evaluated using clinical data of dog patients in a veterinary clinic, as well as in a focus group of experts.

**Abstract:**

Canine ADHD-like behavior is a behavioral problem that often compromises dogs’ well-being, as well as the quality of life of their owners; early diagnosis and clinical intervention are often critical for successful treatment, which usually involves medication and/or behavioral modification. Diagnosis mainly relies on owner reports and some assessment scales, which are subject to subjectivity. This study is the first to propose an objective method for automated assessment of ADHD-like behavior based on video taken in a consultation room. We trained a machine learning classifier to differentiate between dogs clinically treated in the context of ADHD-like behavior and health control group with 81% accuracy; we then used its output to score the degree of exhibited ADHD-like behavior. In a preliminary evaluation in clinical context, in 8 out of 11 patients receiving medical treatment to treat excessive ADHD-like behavior, H-score was reduced. We further discuss the potential applications of the provided artifacts in clinical settings, based on feedback on H-score received from a focus group of four behavior experts.

## 1. Introduction

According to the American Psychiatric Association, Attention-Deficit/Hyperactivity Disorder (ADHD) is defined as persistent symptoms of inattention and/or hyperactivity-impulsivity which interfere with development and/or functioning. Recent surveys estimate the prevalence of ADHD among children within 1–12% (see, e.g., in [1,2]). ADHD is further often associated with abnormalities in social behaviour [3]; enhanced aggression [4]; difficulties of adapting to norms [5]; and cognitive, language, motor, emotional, and learning impairments [6].

ADHD is commonly assessed and diagnosed by relying on information from interviews, observations, and ratings collected from multiple sources (parents, teachers, etc.). Such subjective measures are associated with the risk of informant biases [7] and often present inconsistencies [8]. There is, therefore, increasing interest in objective measures for the diagnosis and assessment of ADHD, in the form of neuropsychological tests [9] and direct measurement of movement [10].

In veterinary medicine, ADHD-like behaviors have been extensively described in domestic dogs (*Canis familiaris*) [11,12,13]. They also have been described as overactivity or hyperactivity [14], hyperkinesis [15], hypermotricity [16], hyperreactivity [17], impulsivity [18], or hypersensitivity-hyperactivity (HSHA) syndrome [19]. This disorder, the prevalence of which is estimated to be between 12 and 34% [14,20,21], is of a special concern due to its being one of the main reasons for dogs’ abandonment [22,23] or even euthanasia [24].

Assessment ADHD-like canine behavior in dogs is much less explored as compared to human ADHD assessment. There are several questionnaires measuring general canine behavior and temperament traits, indirectly addressing inattention and/or hyperactivity-impulsivity through some of their components, such as the Canine Behavioral Assessment and Research Questionnaire (C-BARQ) [25], the Monash Canine Personality Questionnaire-Revised (MCPQ-R) [26], or the Dog Personality Questionnaire (DPQ) [27]. Three assessment tools focus directly on ADHD-like behaviors in dogs: the Dog-ADHD rating scale [11,12,28], the Dog Impulsivity Assessment Scale (DIAS) [18], and the Hypersensitivity-Hyperactivity (HSHA) clinical score [24]. They are owner-administered (and thus inherently subjective) and are not intended for clinical assessment. *Objective* methods for assessment of ADHD-like behaviors in dogs have, to the best of our knowledge, not yet been explored.

Both the genetic and behavioral correlates of inattention and/or hyperactivity-impulsivity have been recently shown to have similarity in humans and dogs. For instance, low levels and poor regulation of serotonin and dopamine are associated with the disruptive and/or violent behaviors exhibited in ADHD in both humans [29] and dogs [30], and a polymorphism of the dopamine receptor (DRD4) gene is a genetic underpinning for this disorder in both humans [31] and dogs [32,33]. Therefore, dogs have been recently highlighted in the literature as a relevant model for human ADHD [12,13,32]. Promoting our understanding of ADHD-like behavior is therefore of increasing interest for both human and veterinary medicine.

Promoting objective measures for the assessment of behavior in general, and of behavioral disorders in particular, is an important challenge for the assessment and diagnosis of both human and canine behavioral disorders.

In the context of human ADHD assessment, objective measures that have been considered can take the form of neuropsychological tests, where the tested person is asked to perform some tests. A similar paradigm has been applied to dogs using touchscreens, supporting them as a model of the association between behavioral disinhibition and ADHD-like behaviors/symptoms [34]. However, this requires some training and special equipment and may not be applicable in clinical settings. Another natural way forward is direct observation of behavior, e.g., in the consultation room.

The goal of this research is to explore objective measures that can be used for the assessment of canine ADHD-like behavior. Our starting point is looking at video recording of the dog’s behavior in the consultation room. One reason is this setup is cheap and feasible to install in any clinic. However, even more importantly, as canine ADHD-like behavior has been reported to be expressed as impulsive and inattentive [11,19,24,35], it is often exhibited in the form of restless and erratic movement around the room, at high speed and taking various angles (as discussed in [36]). Using automatic approaches for tracking, the dog also allows for applying machine learning methods on the obtained time series data, which has the potential to provide useful information about the way the dog moves in the consultation room, interacts with objects or reacts to stimuli.

We specifically address the following research questions:

RQ1 How can we objectively differentiate between dogs with ADHD-like behavior (that requires clinical treatment), and normal controls?
RQ2 How can we objectively assess the degree of dogs’ ADHD-like behavior (that may require clinical treatment)?
RQ3 How can such artifacts inform the design of automatic support for experts’ decision making in clinical contexts?

## 2. Materials, Tools and Methods

Before conducting the study, we captured explicit consent of the dog owners to participate in the study. The procedure was designed with, and approved by, two behavioral veterinarians, in line with published guidelines for the treatment of animals in behavioral research and teaching [37]. Recordings were made as part of regular scheduled veterinary visits. Dogs were allowed to withdraw from the participant at any moment and were not forced to engage with participants.

For automatically tracking the dog movement, we used the Blyzer system, which is described in further details in Appendix A. On top on the tracking module of the system, we implemented a feature computation module (in Python), as explained in Section 2.2 below.

### 2.1. Data Collection

To address our research questions, we collected video data during behavioral consultations in two veterinary clinics at the ‘Veterinar Toran’ Hospital in Tel Aviv and Petach Tikva, Israel. The participating dogs were of two types: normal controls, who arrived to the clinic for standard checkup and/or vaccination procedures, and dogs with excessive ADHD-like behavior who received medical treatment due to this problem. These dogs were recorded in two situations:

- Exploration Trial: free exploration of the room: when entering the consultation room, dog was discharged off leash and left to freely explore the room.
- Dog–Robot Interaction Trial: 20 min into the consultation, the dog was presented a moving dog-like robot, and was left to freely interact with it.

**Figure 1:**
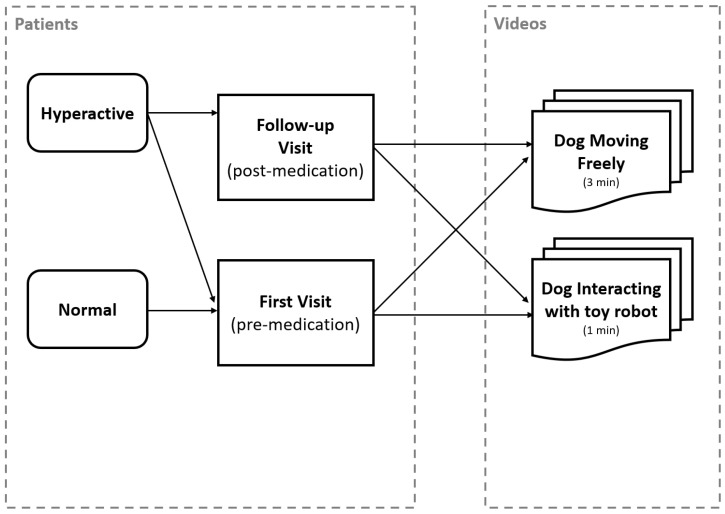
Data collection overview.

The dogs treated for ADHD-like behavior were recorded at two points in time: in their first visit, and follow-up visit after receiving medical treatment. The control group dogs were only recorded once. The process of data collection is presented in Figure 1. In what follows, we provide further details on the participants, location, stimulus (robot), and preprocessing of video recordings.

#### 2.1.1. Location

**Figure 2:**
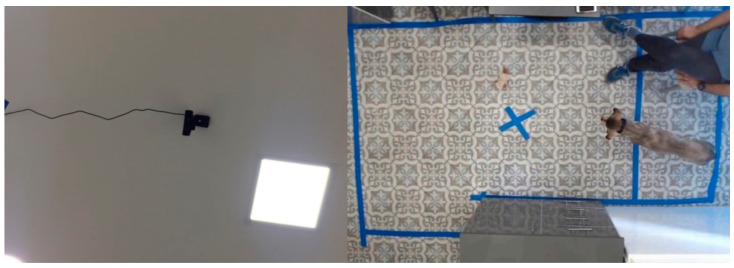
Web camera fixed on ceiling and an example frame. Photos from earlier research capturing video recordings used for the present work [38].

The consultation rooms’ floor sizes (To rule out confounding effects from the difference in floor size in the different clinics, we verified whether there was any significant difference in any measured variable between recording from the Tel Aviv (N = 28) and Petah Tikva (N = 10) clinic. A two-tailed Mann–Whitney U test found no significant difference for any of the variables (*p* > 0.05).) were 260 × 160 (Petach Tikva) and 340 × 220 cm (Tel Aviv), where video was captured by a web camera (Logitech HD Pro Webcam C920) fixed on the ceiling (see Figure 2) and connected to the vet’s computer. During the recording, the vet and the dogs’ owner(s) sat at a fixed location outside of the captured frame.

#### 2.1.2. Robot

**Figure 3:**
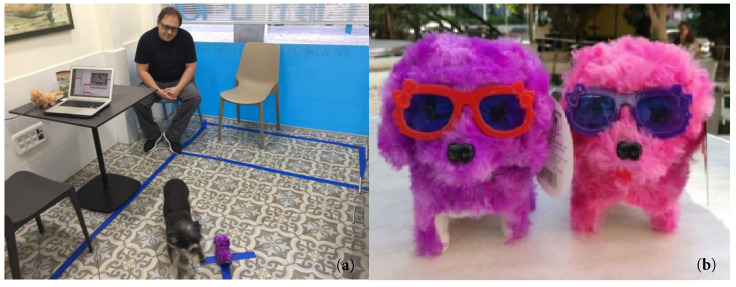
(**a**) Owner and his dog in consultation room in Tel Aviv Clinic. (**b**) Dog-shaped toys used in the experiment. Photos from earlier research capturing video recordings used for the present work [38].

We used a simple commercial dog-shaped toy robot of size 10 cm × 14 cm × 6 cm (see Figure 3b), which made repeated circular movements and barking noise. The latter was disabled by removing the robot’s vocalization mechanism. The robot was placed in a fixed location (marked by X in Figure 2 right and Figure 3a) during veterinary examination.

#### 2.1.3. Participants

Table A1 in the Appendix B presents the participants’ demographic data and the information on their respective recorded trials, as well as their descriptive statistics.

Participants 1–19 formed the H-group, according to the following inclusion criteria:

1. Their first recorded visit was their first visit to the clinic in the context of ADHD-like behavior complaints.
2. The patient was diagnosed with excessive ADHD-like behavior by the consulting behavioral veterinarian.
3. The veterinarian prescribed a medical treatment (with or without addition of behavior correction) for treating excessive ADHD-like behavior.

Participants 20–38 formed the C-group, which included dogs with no reported health issues, visiting the hospital for annual checkup or vaccination. During their consultation, the behavioral vet ruled out any behavioral-related disorder and other comorbidities.

#### 2.1.4. Trial Protocols

As mentioned above, the participants had two trials: (i) exploration and (ii) interaction with toy robot. For the first part, owner and dog entered the room simultaneously, the owner took place in the designated chair, the vet sat by his desk. The owner(s) took their place at a predefined spot in the room. They were requested not to interact or make eye contact with the dog during the experiment, regardless of what the dog was doing. Video recording (Recording samples can be found here (exploration trial) and here (dog–robot interaction)) was started (the vet and the owner are outside of camera scope, and only the dog and the robot are visible on the recording). The dog was allowed to freely move around the room while the veterinarian interviews the owner(s), also filling out information on his computer. The veterinary doctor and the owner(s) were always placed in the same location (fixed chairs in the room), except for the moment at which the robot was introduced in the middle of the room. An owner with his dog is shown in Figure 3. At this point the dog was released off leash, and video recording started.

The second part had the following structure (A similar protocol was used in an earlier work [39]). The dog was brought into the room and taken off its leash. Introduction phase: About 20 min into the interview, the veterinarian placed the inactive robot in the center of the room and returns to his chair. The dog was recorded for three minutes. Testing phase: The veterinarian activated the robot and returned to his place. Interaction of the dog with the moving robot was recorded for three minutes. The veterinarian then deactivated the robot and put it away. The dog was recorded for additional 10 min after the end of testing phase. The introduction phase was introduced in order to let the dog get acquainted with a strange object, thus preventing too high stress levels of patient dogs.

#### 2.1.5. Video Recordings Processing

**Figure 4:**
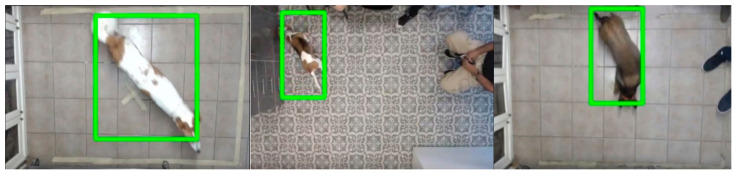
Frames example of dogs being tracked by Blyzer.

**Figure 5:**
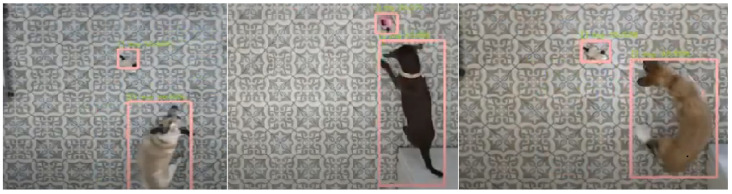
Frames example of dogs and robots being tracked by Blyzer.

**Figure A1:**
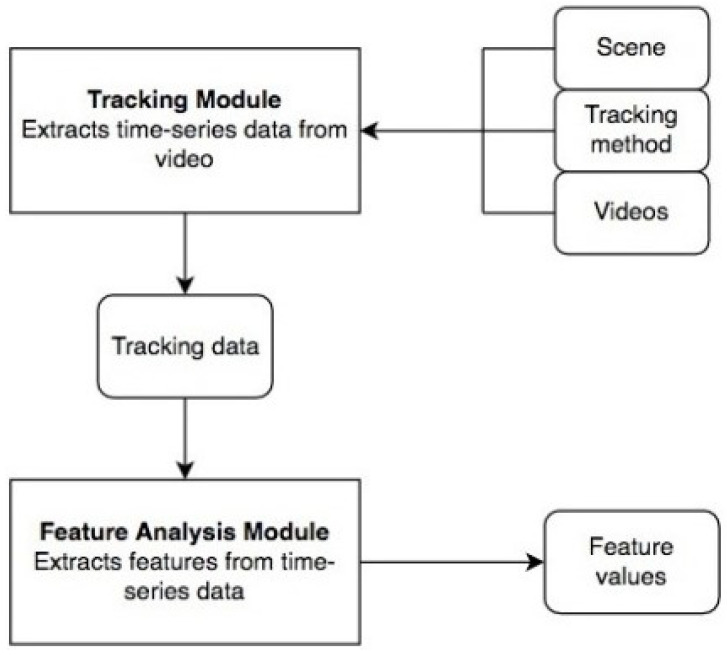
BLYZER Architecture.

*Automatic tracking.* The automatic tracking module of Blyzer was run on the videos. The tracking method (neural networks) used the following elements (see also Figure A1 in the Appendix A). For the exploration trial, we used a neural network based on the FASTER RCNN architecture [40] pretrained on COCO and Pascal Voc datasets, in addition to 6000 annotated frames from our vet clinics dataset. Figure 4 shows example frames where the dog object is detected. For robot detection we used the MobileNets SSD framework [41], pretrained on COCO, Kitti, Open Image, AVAv2.1, iNaturalist and Snapshot datasets, in addition to 550 annotated frames from the vet clinic dataset. Figure 5 shows example frames with dog and robot detection. Postprocessing operations supported by Blyzer (such as smoothing and extrapolation) were applied to remove noises and enhance detection quality.

*Filtering of low-quality tracking.* The following inclusion criteria for videos were defined for both types of trials: (i) percentage of frames where dog is present is at least 70% of the frames, and (ii) dog and robot are identified with average certainty threshold above 70%. In Table A1 videos excluded using these criteria are marked with ‘-’.

### 2.2. Choice of Features

In Blyzer architecture (see Figure A1 in Appendix A), the feature analysis module is responsible for extracting the values of higher-level *features*, in our context they are related to the dog’s movement trajectory, and its interaction with robot. Thus, we needed to add to the library the implementation of features which are relevant for our problem and domain.

A feature in machine learning (ML) is an individual measurable property of what is being observed [42]. Many different features can be extracted in this case, however not all of them may be meaningful or relevant for our problem and domain. One possible way forward is using standard feature extraction and selection strategies [42]. Another alternative is by relying on expert knowledge to manually select the promising features. Due to the exploratory nature of our study, we combined these approaches in the following way. First, we held in-depth interviews with experts, and performed a literature review related to metrics of animal movement trajectories. After compiling a list of potential features, we applied four different feature selection techniques, which yielded four different subsets of features suggested for use by classification algorithms. Below we describe this process and the obtained features in further details.

1. *Expert interviews.* For elicitation of possible features from experts, we held in-depth semistructured interviews with four behavioral specialists. (One was Dip. ECWABM, one was ECWABM resident, one was veterinary doctor consulting on behavior, and one was a dog trainer and a researcher (PhD) in dog behavior.) During interviews, we first asked them to characterize (i) free movement of a dog with excessive ADHD-like behavior, and (ii) interaction of such dog with a toy robot, as opposed to a dog with no such problem. Appendix C provides the details on the chosen features. Table A2 summarizes behavioral notions mentioned by the experts, and their characteristics for the two types of dogs, as well as their mapping to potential features. Table A3 presents a list all the chosen features which are also explained in further details.
2. *Animal movement metrics.* The description of animal movement paths is also a cornerstone of movement ecology [43]. A common characteristic used to describe and analyze movement paths is *tortuosity*, or how much tortuous and twisted a path is. We hypothesized tortuosity can be related to the experts’ highlighting ‘erratic movement’ and ‘turning around’ (Table A2). Thus, we selected as features the following five movement indices, which have been linked to tortuosity in [44]: straightness, Mean Squared Displacement, Intensity of Use, Sinuosity, and Fractal D; Table A4 provides their mathematical definitions and references.
3. *Feature Subset Selection.* Feature selection involves analyzing the relationship between input variables and the desired variable while selecting those input features that have the highest correlation with the target variable. Two of the most commonly used feature selection methods types (i) filter-based methods, which select subset of features based on their correlation with the target feature, and (ii) wrapper-based methods, which search for well-performing subset of features [45,46,47]. We chose to apply three filter-based algorithms: Univariate Correlation (f-classif), Chi${}^{2}$ and Importance, and one wrapper-based: Recursive Feature Elimination (RFE). Table A5 presents the results of selections made by each of these two methods for two trials: E (exploration) and DR (dog–robot) (The reason we separated the two was because the set of dogs who had both trials available was smaller than the set of dogs who had only the exploration trial.).

### 2.3. Classification Models and the H-Score

We experimented with several well-known classification algorithms: stochastic gradient descent, random forest, k-nearest neighbors, gaussian process, gaussian naive bayes, multinomial naive bayes, bernoulli naive bayes, complement naive bayes, and support vector machines [48]. Each of these algorithms was run with each of the subsets of features suggested in Table A5 in the Appendix C.

We used leave-one-out cross-validation, which is a standard method for evaluating the performance of classification algorithms [49]. We further used the following classification accuracy metrics: precision, recall, F-measure, and ROC. Precision and recall use the notions of True Positive (*TP*), False Positive (*FP*), False Negative (*FN*), and True Negative (*TN*). *TP* and *FP* refer as correct/incorrect positive prediction (that the dog is hyperactive), while *FN* and *TN* refer to correct/incorrect negative prediction (that the dog is in control group).

Precision (*P*, or specificity) and Recall (*R*, or sensitivity) are defined as follows:

$$P=\frac{N_{TP}}{N_{\left(TP+FP\right)}}\;\;\;R=\frac{N_{TP}}{N_{\left(TP+FN\right)}}$$

The F-measure (also called $F_{1}$) represents the combination of precision and recall:

$$F_{1}=\frac{2PR}{P+R}$$

ROC curve-based metrics provide a theoretically grounded alternative to precision and recall. The ROC model attempts to measure the extent to which an information filtering system can successfully distinguish between signal (relevance) and noise [50].

To provide the H-score which would assess the level of ADHD-like behavior, we decided to look at class probabilities offered by the different models.

### 2.4. Focus Group of Experts

To evaluate the H-score and the whole approach of objective assessment in a clinical context, we conducted a semistructured Focus Group Discussion (FGD) [51] to explore the perceived usefulness of the objective hyperactivity assessment, and elicit any further usability requirements.

As the quality of FGD data relies heavily on the selection of appropriate participants and targeted questions [52], with only a few focus groups typically sufficient to achieve data saturation [53], we opted for a maximum stratification approach by including experts from different (dog-related) backgrounds, with different levels familiarity of computer-aided diagnostic systems. This led to a selection of four total participants: three behavioral veterinarians, one of which had prior experience with computational animal behavior analysis systems, and one animal behavior researcher with expertise in dog training.

The FGD was structured as follows:

- Participants were welcomed by the moderator, and the purpose of the FGD was explained.
- Participants were asked to discuss (i) the use of ML for objective behavior assessment, and (ii) the use of ML for assessment of ADHD-like behavior within their professional practice.
- Next, we showed:
  An example of exploration trial of a normal dog and of a hyperactive dog (see the video here) and presented their respective H-scores.
  Two examples of exploration trials of a hyperactive dog before and after clinical treatment (see the video here) and presented their respective H-scores.
- We next asked participants to discuss:
  To what extent they felt the H-score was consistent with their own expert opinion on the watched video;
  To what extent they felt the H-score would support them in clinical practice, and how;
  To what extent they felt using the H-score would be well integrated in clinical practice.

We used follow-up questions in order to elicit additional information, triggered by mentions relating to specific non-functional requirements such as the speed of analysis, security aspects, etc.

The FGD session was conducted over Zoom. We live transcribed and took notes during the session, which we then discussed and analyzed in order to determine key reactions from the FGD participants.

## 3. Results

### 3.1. Hyperactivity Classification Results (RQ1)

Out of all the options we experimented with, the Random Forest classification algorithm achieves optimal results (83.3% precision, 80% recall, 81% F1-score, and 81.6% ROC score). The details of the comparison as well as the list of the prevalent features are presented in Appendix D.

### 3.2. H-Score Evaluation Results (RQ2)

The H-score was taken as the class probability of the classification model. Table 1 presents the H-scores of the H-group, together with information on the recommended treatment, behavioral modification was also suggested (B.mod column). Eleven participants from the H-group had also a follow-up visit (after receiving medical treatment), time passing between the visits (in months) appears in column TbV. For them, we compared the H-scored between the first and follow-up visit: as can be seen, in 8 out of 11 patients the H-score was reduced. The three dogs in which it was not reduced (but stayed the same or increased) were dogs who indeed have not shown sufficient progress in the vet’s opinion, as further medication was prescribed in the follow-up visit.

Table 2 further shows the H-scores of C-group participants.

When comparing the H-scores of the first visit between C-group (N = 19) and H-group (N = 19), the C-group score was found to be significantly lower (median = 0.26) than that of the H-group (median = 0.96) (two-tailed Mann–Whitney U = 49.5, *p* < 0.00001).

### 3.3. The H-Metric in Clinical Context (RQ3)

Upon showing them comparative recordings of pre- and post-treatment phases, overlaid with H-metric scores, all focus group participants agreed with the observed difference in hyperactivity scores.

Based on our analysis of the focus group discussion, we conclude that the H-score is perceived by behavioral experts as a valuable tool in the context of assessment of symptoms of ADHD-like behavior in the context of clinical treatment. This is due to its complete objectivity, as opposed to all other assessment methods available today in the context of ADHD-like behavior. Yet, the experts noted that clinical diagnosis cannot be based solely at the H-score, and additional information is required. This also explains why the participants found the accuracy of the tool satisfactory, claiming one should not expect higher accuracy of the classification models with the present dataset looking only at the first three minutes of the dog’s behavior.

The H-score also is perceived as useful for communication of treatment outcome to the dog owner. Outside of clinical context, as a side note, it was noted that the tool also has potential for preventive alerts to owners about the potential ADHD-like behavior of their dogs, if in the future it is implemented as a tool for owners and not only for clinical experts.

Further details concerning the analysis of the focus group discussions can be found in Appendix E.

## 4. Discussion and Future Research

In this study, we introduced a novel method for assessing canine ADHD-like behavior using machine learning techniques. The method is completely *objective*—it analyzes movement of dogs based on a video footage and without relying on (potentially subjective) information from owner or the vet. However, the latter is also in some sense a limitation of the method in its ability to support diagnostic decision-making, as it may not take into account critical information which is not observed in the video.

We explored the potential of such approach to classify excessive indications of ADHD-like behavior, and to quantify its degree. We have found that the Random Forest classification algorithm reached the best performance (with 83.3% precision, 80% recall, 81% F1-score, and 81.6% ROC score). The most prevalent features were found to be total distance and average speed, reflecting the intuition of erratic movement around the room, expressed in expert interviews.

We further explored the perceptions of behavioral veterinarians on the usefulness and feasibility of this approach in clinical settings using a focus group. The experts agreed on the potential of a tool offering objective measurement of symptoms of ADHD-like behavior in the context of their clinical practice, and also agreed that perhaps much better cannot be achieved, due to obvious lack of important information (such as background information about the dog, or its environment) in the short footage analyzed.

Due to the exploratory nature of this research, we faced some major challenges, and had to make concrete decisions related to the design of this study, and its potential threats to validity, which we discuss below.

Data collection in a consultation room of an animal hospital entails that the setting is not completely controlled. To mention just some aspects which may have affect on the dog’s behavior: scents and noises outside the consultation room, time of visit, and what the dog experienced prior to visit. To mitigate these threats, we made sure that the places where the vet and owner(s) sat were always fixed, using marking on the floor. We also excluded from the dataset consultations in which another veterinarian entered the room and interrupted the standard protocol, or the owner went out, leaving the dog alone in the room.

The use of Blyzer’s deep learning models for object detection made the processing of a whole consultation (approximately 40 min) infeasible in terms of processing times, and decisions *which* fragments to analyze were also crucial. After consulting with several behavioral experts, it was decided that the first three minutes of the visit are of crucial importance, as they introduce the dog into a novel environment, and its reaction at the first minutes is the most informative. Some participants of the focus group also remarked on including additional video footage, e.g., from the dog’s home as being potentially important. However, this poses challenges due to the non-uniform shooting angle and room size, as well as the complete inability to control the dog’s environment. Based on an earlier study [39] which used dog–robot interactions as a tool for eliciting reactions from dogs in the context of a behavioral problem, we decided to also add such dimension to the considered protocol. However, the obtained final model which had the best performance did not make use of any of the features of the dog–robot interaction. This could indicate that the first three minutes are more informative in the context of ADHD-like behavior. However, note that the number of dogs at which we looked in the context of dog–robot interactions was smaller than the overall number due to technical reasons (low-quality videos being filtered out), this too could be an explanation for these features ending up not being included, so this issue needs further examination with a larger dataset.

Reflecting further on practical aspects of using the suggested approach in clinical settings, it is important to note that in addition to the high processing time needed to produce the tracking data (which can be addressed by using stronger machines), another problematic aspect with which we faced in our study was quality of data. This can be divided into two dimensions: (i) quality of detection when dog is in frame and (ii) quality of footage with the dog going out of frame too frequently. Item (i) can be addressed by improving the tracking models used by extending their training set to include more dogs of different sizes, colors, and breeds. Item (ii) was mainly by privacy considerations, as the owner needed to be left out of frame. This could be partially addressed by using more sophisticated interpolation techniques, predicting the dog’s movement even when it is not visible. However, it is clear that these considerations need to be taken into account when planning a tool that will provide real-time (or near real-time) H-score in a consultation room that would be integrated in the clinician’s workflow.

Another limitation of this study is the rather limited number of dogs in our dataset. This is related to the fact that we decided to recruit participants who only exhibited pure ADHD-like symptoms without comorbidities. Re-examination of our results with a significantly larger dataset is a natural step for further research.

A further direction for future research is considering other behavioral disorders than ADHD-like behavior, as well as ADHD mixed with further comorbidities such as anxiety, depression, etc. These may call for changes in the selected features, which need to be elicited in further interviewing experts concerning the specific way in which these conditions are reflected in the dog’s behavior and/or its interaction with humans or objects.

Based on the focus group findings, the suggested approach seems promising in the context of clinical decision making of behavioral veterinarians, as well as for non-clinical behavior assessment of canine professionals, as it offers an *objective tool* which is much appreciated in behavior assessment which is usually based on subjective reports, or owner-filled questionnaires. An important aspect for future research is the role social cues play in eliciting hyperactive behavior. Extending our approach using protocols which integrate social cues (such as hand gestures, looking at the dog, petting the dog, etc.) are an important direction for future research on objective assessment of ADHD-like behavior. 

## Figures and Tables

**Table 1:** H-score of two (first and follow-up) visits of H-group.

ID	Dog Name	Consultation	H-Score	Medication	B. mod.	TbV
1	Pery	First	0.73	Fluoxetine 80 mg	+	
1	Pery	Follow-up	0.01			2
5	Indi	First	0.91	Fluoxetine 60 mg	-	
5	Indi	Follow-up	0.82			2
6	Dafi	First	0.96	Fluoxetine 50 mg	+	
6	Dafi	Follow-up	0.25			2
7	Bana	First	0.7	Fluoxetine 50 mg	-	
7	Bana	Follow-up	0.26			2
16	Kim	First	0.97	Fluexetine 60 mg	+	
16	Kim	Follow-up	0.67			1
18	Henri	First	0.97	Fluoxetine 20 mg +	+	
				Trazodone 25 mg		
18	Henri	Follow-up	0.86			2
4	Humus	First	0.2	Fluoxetine 70 mg	+	
4	Humus	Follow-up	0.02			2
12	Nancy	First	0.25	Fluoxetine 60 mg	+	
12	Nancy	Follow-up	0.01			2
10	Lichi	First	1	Fluoxetine 60 mg	+	
10	Lichi	Follow-up	1	Fluoxetine 70 mg+		2
				Cyproterone Acetate 100 mg		
14	Angy L.	First	0.99	Fluoxetine 40 mg	-	2
14	Angy L.	Follow-up	0.99	Fluoxetine 40 mg		
11	Tomy	First	0.45	Fluoxetine 40 mg +	-	2
				Cyproterone Acetate 50 mg +		
11	Tomy	Follow-up	0.54	Fluoxetine 40 mg +		
				Cyproterone Acetate 50 mg		
2	Patrick	First	0.98	Fluoxetine 90 mg	-	
3	Delpi	First	0.36	Fluoxetine 80 mg	+	
8	Guizmo	First	1	Fluoxetine 40 mg	-	
9	Max	First	0.63		-	
13	Angy K.	First	0.89	Fluoxetine 30 mg	-	
15	Pit	First	1	Fluoxetine 80 mg	+	
17	Sia	First	1	Fluoxetine 60 mg	+	
				Trazodone 75 mg		
19	Mitch	First	1	Fluoxetine 40 mg	-	

**Table 2:** H-score of the (single) visit of C-group.

ID	Dog Name	H-Score
20	Bella	0.34
21	Dream	0.1
22	Gino	0.86
23	Brutus	0.08
24	Waaly	0.02
25	Theresa	0.26
26	Belle	0.35
27	Jema	0.01
28	Laila	0.05
29	Ketem	0.26
30	Sparki	0.22
31	Boby	0.07
32	Ringo	0.42
33	Mika	0.87
34	Pie	0.02
35	Mila	0.61
36	Chelsee	0.25
37	Pachita	0.63
38	Pit.	0.99

**Figure A2:**
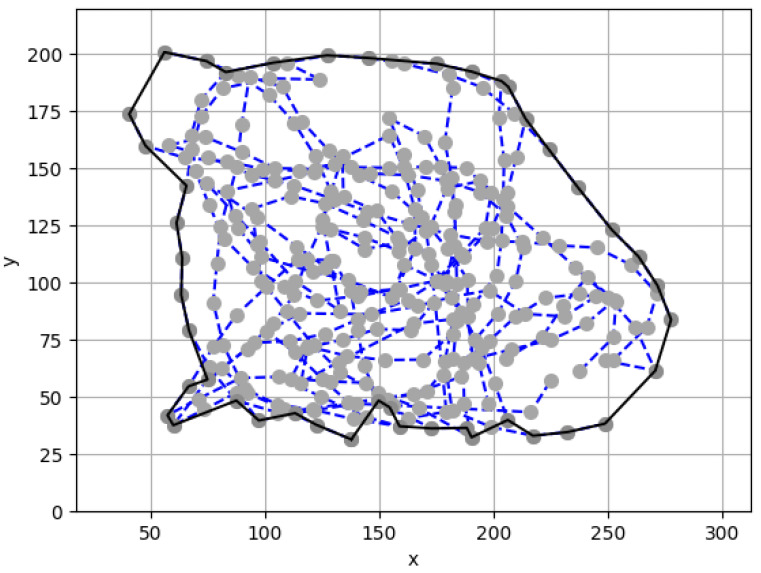
Number of Points visualization example.

**Figure A3:**
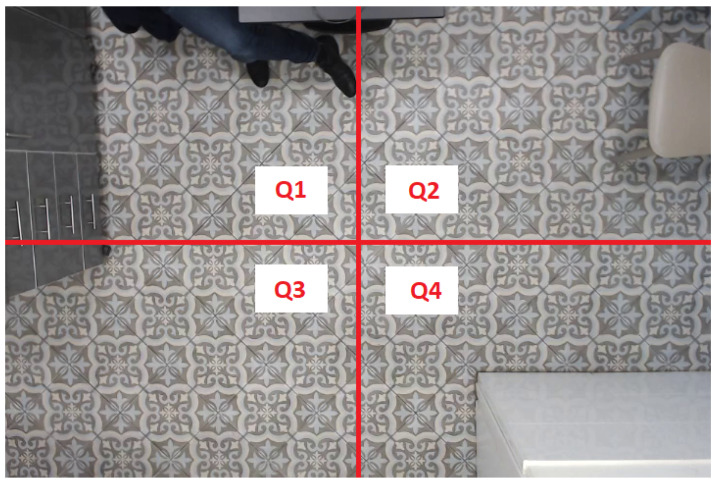
Consultation room divided into quadrants.

## Appendix A. The Blyzer System

The Blyzer system [36,38,54] aims to provide automatic analysis of animal behavior with minimal restrictions on the animal’s environment (unlike tracking systems designed for rodents, e.g., in [55], which are usually situated in a semi-controlled restricted setting), or camera setting (as opposed to, e.g., the works in [56,57] where a 3D Kinect camera is used).

Blyzer’s input is video footage of a dog freely moving in a room and possibly interacting with objects, humans, or other animals. Its output includes measurements of specific parameters specified by the user, which then provide some form of quantification of behavioral parameters.

Blyzer has already been used for a number of animal behavior projects. One example is a multi-method study, combining fMRI, eye-tracking, and behavioral measures, where Blyzer was utilized for the latter purpose to explore the possibility of a neural attachment system in dogs. Full details are provided in [58].

Another example is the analysis of time budget and sleeping patterns of breeding stock kenneled dogs as welfare indicators. The dogs, bred and maintained by the Animal Science Center in Brazil, were observed for eight consecutive months using simple security cameras installed in their kennels (using night vision at night). Blyzer was used to measure parameters such as total amount of sleep, sleep interval count, and sleep interval length, for further details see in [36].

The most relevant use of Blyzer to our purposes is the study in [39], where a setting similar to ours—recording in the consultation room of a behavioral veterinarian—was used in the context of a different behavioral issue related to anxiety. This study makes use of the idea of using artificial agents as stimuli to elicit responses from a dog. In particular, various robots have been used for studying canine social behaviors. For example, Leaver and Reimchen [59] investigated the approach preference of dogs towards a dog-like robot with different tail sizes and movements. Gergely et al. [60] examined dogs’ interactive behavior in a problem-solving task in which the dog had no access to the food with three different social partners, two of which were simple robots (remotely controllable cars), and the third was a human behaving in a robot-like manner. Dogs’ interactions with more complex commercial robots, displaying a wide variety of (programmed) behavior and/or similarity to the target species, have also been explored [61]. Given that dogs exhibit social behaviors towards robots, this study’s hypothesis was that canine behavioral disorders that are related to social fear, may also be reflected in the way dogs interact with robots. Thus, the use of dog–robot interactions (DRIs) was examined as a tool for the assessment of canine behavioral disorders. An exploratory study, recording DRIs for a group of 20 dogs, consisting of 10 dogs diagnosed by a behavioral expert veterinarian with deprivation syndrome, a form of phobia/anxiety caused by inadequate development conditions, and 10 healthy control dogs. It was found that pathological dogs moved significantly less than the control group during these interactions, thus confirming the hypothesis. This provided an inspiration for our study, where we also analyzed DRIs in the context of ADHD-like behavior.

## Appendix B. Participants’ Details

Table A1 presents the details of participants from the two groups: The C-Group (N = 19, 8 males, 11 females) and the H-Group (N = 19, 10 males, 9 females).

**Table A1:** Participant demographics and trial information.

ID	Patient Name	Breed	Weight	Age	Sex	Neutered	Group	First Visit			Second Visit		
								**Rec.**	**E.T.**	**DR.T.**	**Rec.**	**E.T.**	**DR.T.**
1	Pery	English Bulldog	21	5.0	M	Y	H	+	+	+	+	+	-
2	Patrick	Husky	23	0.6	M	N	H	+	+	-	-	NA	NA
3	Delpi	Mixed	34	2.0	M	Y	H	+	+	-	-	NA	NA
4	Humus	Mixed	23	1.5	M	Y	H	+	+	-	+	+	No
5	Indi	Vizsla	20	1.5	F	Y	H	+	+	-	+	+	+
6	Dafi	Mixed	24	2.0	F	Y	H	+	+	+	-	NA	NA
7	Bana	Doberman	32	1.5	F	Y	H	+	+	+	+	+	-
8	Guizmo	Mixed	13	4.5	M	Y	H	+	+	-	-	NA	NA
9	Max	Labrador	36	1.0	M	Y	H	+	+	-	-	NA	NA
10	Lichi	Mixed	22	7.0	F	Y	H	+	+	+	+	+	-
11	Tomy	French Bulldog	13	2.5	M	Y	H	+	+	-	+	+	-
12	Nancy	Mixed	21	0.8	F	Y	H	+	+	+	+	+	+
13	Angy K.	Mixed	26	3.0	F	Y	H	+	+	+	-	NA	NA
14	Angy L.	Beagle	25	2.5	F	Y	H	+	+	-	+	+	+
15	Pit	Mixed	24	1.0	M	Y	H	+	+	+	+	+	-
16	Kim	Mixed	18	1.0	F	Y	H	+	+	+	+	+	+
17	Sia	Mixed	19	2.0	F	Y	H	+	+	+	+	+	-
18	Henri	Jac Russel	18	1.0	M	Y	H	+	+	+	+	+	-
19	Mitch	French Bulldog	13	6.0	M	N	H	+	+	-	-	NA	NA
20	Bella	Mixed	8	3.0	F	Y	C	+	+	+	NA	NA	NA
21	Dream	Golden Ret.	35	10.0	F	Y	C	+	+	-	NA	NA	NA
22	Gino	Cane Corso	44	0.7	M	N	C	+	+	+	NA	NA	NA
23	Brutus	Bullmastif	29	2.5	M	N	C	+	+	-	NA	NA	NA
24	Wally	Saluki	23	3.0	M	Y	C	+	+	-	NA	NA	NA
25	Theresa	Saluki	16.5	0.8	F	Y	C	+	+	+	NA	NA	NA
26	Belle	Mixed	23	4.0	F	Y	C	+	+	+	NA	NA	NA
27	Jema	Mixed	25	4.0	F	Y	C	+	+	+	NA	NA	NA
28	Laila	Mixed	20	1.0	F	Y	C	+	+	-	NA	NA	NA
29	Ketem	Mixed	16	10.0	F	Y	C	+	+	-	NA	NA	NA
30	Sparki	Golden Ret.	40	5.0	M	N	C	+	+	+	NA	NA	NA
31	Boby	Mixed	42	4.5	M	Y	C	+	+	-	NA	NA	NA
32	Ringo	Mixed	25	4.5	M	Y	C	+	+	-	NA	NA	NA
33	Mika	Mixed	7	7.0	F	Y	C	+	+	+	NA	NA	NA
34	Pie	Mixed	25	1.0	M	Y	C	+	+	+	NA	NA	NA
35	Mila	Mixed	22	3.5	F	Y	C	+	+	-	NA	NA	NA
36	Chelsee	Mixed	40	5.0	F	Y	C	+	+	-	NA	NA	NA
37	Patchita	Mixed	13	8.0	F	Y	C	+	+	+	NA	NA	NA
38	Pit	Mixed	25	3.0	M	Y	C	+	+	+	NA	NA	NA

**Remark** **A1.**
*To avoid discriminating effects of demographic factors during the research, we ensured that both H-Group and C-Group will hold cohesive demographic factors. To confirm this, the two groups’ demographic factors showed no statistically significant differences: (i) weight did not differ significantly between the two groups (Mann–Whitney U test, U = 128, TA = 28 & PT = 10, p = 0.64 (two-tailed)), (ii) age (Mann–Whitney U test, U = 111, TA = 28 & PT = 10, p = 0.34 (two-tailed)), (iii) sex (Mann–Whitney U test, U = 97, TA = 28 & PT = 10, p = 0.09 (two-tailed)), and (iv) neutered state (Mann–Whitney U test, U = 111, TA = 28 & PT = 10, p = 0.34 (two-tailed)), (iii) sex (Mann–Whitney U test, U = 108, TA = 28 & PT = 10, p = 0.07 (two-tailed)) did not differ significantly between the two groups.*

**Remark** **A2.**
*Note that while Table A1 refers to two visits of each participant to the clinic, the second (follow-up) visit is only relevant for dogs from the H-group. Moreover, dogs participating in the first round of visits which did not return to the clinic for a follow-up during the time of our study are marked with ‘−’ in the Recording Available column (abbreviated by ‘Rec.’). Only 12 out of 19 participants of the H-group returned for a follow-up visit. Moreover, out of the recorded trials, not all of them were of sufficient quality (our quality metrics are explained below); some of them, marked with ‘−’ in the ‘E.T’ and ‘DR.T’ columns were excluded, as explained in Section 2.1.5 below. Irrelevant fields (follow-up visit for the C-group, or E.T/DR.T fields for unavailable recordings) are marked with ‘NA’.*

## Appendix C. Feature Selection

Table A2 summarizes behavioral notions mentioned by the experts during the interviews in relation to ADHD-like behavior, and their characteristics, as well as their mapping to potential features. Table A3 presents a list all the chosen features which are also explained below in further details—divided into exploration trial and dog–robot interaction trial.

**Table A2:** Experts’ descriptions and mapping to features.

Behavioral Notion	ADHD-Like	Normal	Potential Features
speed of movement	higher	slower	speed
turning around	excessive	moderate	num of turns
exploration	excessive	standard	area, distance
movement around the room	erratic	more ordered	num of points, area
vet and owner proximity	excessive interest to vet	same interest	stay in quadrants
interest to robot	excessive	normal	TFC, DFC
movement to robot	excessive	normal	pace, TL

**Exploration Trial Features:**

**Total distance:** The length of the dog’s movement trajectory.

**Speed:** In a previous study [39] analyzing dog movement in the consultation room in the context of another behavioral problem (anxiety), average speed was used. We also added the standard descriptive statistics of pointwise characteristics of median, maximum, variance and standard deviation (see, e.g., in [62]).

**Number of turns:** To capture excessive turning around the room, we defined the parameter of number of turns, dividing it also to four types according to angle sharpness: between 30–60°, 60–90°, 90–120°, and above 120°. To calculate the degree, the spatio-temporal was divided to vectors, and the degree between vectors was calculated as the inverts-cosine of the distance to intersection-point and shown in the following formula:

$$angle=arccos\left[\frac{\left(\left(x_{2}-x_{1}\right)*\left(x_{4}-x_{3}\right)+\left(y_{2}-y_{1}\right)*\left(y_{4}-y_{3}\right)\right)}{\sqrt{\left({\left(x_{2}-x_{1}\right)}^{2}+{\left(y_{2}-y_{1}\right)}^{2}\right)}*\sqrt{\left({\left(x_{4}-x_{3}\right)}^{2}+{\left(y_{4}-y_{3}\right)}^{2}\right)}}\right]$$

**Area:** To calculate the dog’s area covered during consultation, convex hull is first calculated. Convex hull is the smallest polygon shape that contains the whole vectors traveled by the dog (see the black polygon in Figure A2). The area is the region occupied inside the convex hull polygon and given the ordered vertices that engulf it, Shoelace formula [63] is applied:

$$\mathrm{Area}=\frac{1}{2}|\sum_{i=1}^{n-1}X_{i}Y_{i+1}+X_{n}Y_{1}-\sum_{i=1}^{n-1}X_{i+1}Y_{i}+X_{1}Y_{n}|$$

**Number of points:** To capture the smoothness of the trajectory, we defined the parameter of the number of points found on the curve of the dog’s trajectory, obtained by segmenting and smoothing it. Various filtering techniques can be used to smooth the trajectory (e.g., moving average [64]); we chose a variant (Intuitively, our variant of the Ramer–Douglas–Pecker curve approximation reduces the number of a points in a curved ’polyline’ that is approximated by a series of points by defining a straight line between first and last point in a set of points that form the curved line. It finds the furthest point from this line and checks if its closer than a given distance. If so, it removes all the points while keeping only the first and last. If not, the curve is split as follows: (1) from first line up to, and including, the outlier; (2) from the outlier to the last point. The process is applied iteratively) of the Ramer–Douglas–Pecker curve approximation algorithm [65]. Figure A2 shows example of dog movement’s graph where the points obtained by the above mentioned segmentation are in gray.

**Quadrant point count:** The consultation room was divided into four quadrants of equal size, numbered as in Figure A3. Let $PQ_{i}$ denote the number of points of a dog’s trajectory belonging to quarter *i*.

**Dog–Robot Interaction Trial Features:**

**Time until first contact with robot:** TFC is defined as the time from start of DR-trial to the time when the dog comes to close proximity to robot (using a predefined threshold).

**Duration of first contact with robot:** DFC is defined as the duration of contact for the dog and robot (i.e., the dog being in proximity to robot).

**Trajectory length:** TL is defined as the sum of pixel the dog covered, including the time of interaction with robot.

**Pace:** Pace is defined as the ratio between the length of the trajectory until first contact and the time until first contact.

Table A4 presents the list of movement indices included in the feature list. Below we provide more detailed explanations of these indices.

**Intensity of use:** IU is defined as the ratio between total movement and the square root of the area of movement [66], intensity of use is proportional to the active time spent per unit area, which should increase with tortuosity of the path.

**Straightness:** The straightness ST (or linearity) index is defined as the Euclidian distance between the start and the final point, divided by the total length of the movement [67].

**Sinuosity:** The Sinuosity, SI, assumes that paths are correlated random walks, and thus were produced by animals randomly searching a homogenous environment [68].

**Mean square displacement:** The Mean Square Displacement, MSD, is an important parameter used as index of movement area or home range [69]. It is likely to be inversely related to path tortuosity, similarly to ST, as more tortuous paths take more time to leave a certain area.

**Fractal dimension:** The fractal dimension of a path, D, is another measure of tortuosity that has been used [70], based on the theoretical framework of fractal geometry. The Fractal D of a set of two points (as a curve) can be seen as a measure of its propensity to cover the plane, being a value of one for no plane coverage (a straight line, for example) and two for full coverage of some area in the plane. Generally, Fractal D must be correlated with path tortuosity, but it is more appropriately considered an area-filling index.

**Table A3:** Summary of potential features.

Variable	Explanation	Units
Total distance	Distance covered by the dog	cm
turn30_60	Number of turns between 30 and 60 degrees	
turn60_90	Number of turns between 60 and 90 degrees	
turn90_120	Number of turns between 90 and 120 degrees	
turn120	Number of turns greater than 120 degrees	
area	Polygon area of the dog’s Convex hull movement	cm${}^{2}$
IU	(Intensity of Use) the ratio between total movement and the square root of the area of movement	Percentage
ST	Straightness-net displacement distance divided by the total length of the dog’s movement	Varies from 0 to 1
MSD	Mean squared displacement-measure of the deviation of the position of a particle with respect to the dog’s reference position over time	cm${}^{2}$ s${}^{-1}$
SI	Sinuosity-calculation of the actual path length divided by the shortest path length of the dog’s movement	Varies from 0 to infinity
FD	Fractal Dimension-statistical index ratio of complexity comparing the space-filling capacity of the dog’s movement pattern	
Average Speed	the dog average speed	cm/s
Speed Median	the dog speed’s median	cm/s
Speed Variance	the variance of the dog’s speed	(cm/s)${}^{2}$
Speed stdev	the standard deviation of the dog’s speed	cm/s
Max	The max speed of the dog	cm/s
Number of points	a variant of Douglas–Peuker curve approximation algorithm to the dog’s trajectory	
Quadrant 4 points	Number of points the dog appears in the 4th quadrant	
Time until first contact with robot	Time passed between robot being presented to the dog and the moment of contact between dog and robot	s
Duration of first contact with robot	The duration of dog–robot contact during the first contact	s
Trajectory length	The total distance the dog covered during the video recording	cm
Pace	The ratio between the Trajectory until contact and time until first contact	cm/s

**Table A4:** Movement indices.

Index	Equation	Parameters Descr.	Reference
Straightness (ST)	ST = $\frac{dE}{L}$	dE - Euclidean path distance between two points, L - trajectory length between them. The Straightness S(p1, p2) between two points p1 and p2∈P is defined as their ratio between the Euclidean path distance (dE) and their graph movement (L).	[44,71]
Mean Squared Displacement (MSD)	MSD = VarX+VarY	Where X and Y are Cartesian location coordinates around the movements group centroid	[44,69]
Intensity of Use (IU)	IU = $\frac{L}{\surd A}$	where L is the total path length and A is the movement area	[44,66]
Sinuosity (SI)	SI = $2{\left[p\left(\frac{1-c^{2}-s^{2}}{{\left(1-c\right)}^{2}+s^{2}}+b^{2}\right)\right]}^{-0.5}$	Where p is the mean step length, c is the mean cosine of turning angles s is the mean sine of turning angles and b is the coefficient of variation of step length	[44,68,72]
Fractal D (FD)	FD = $\frac{\theta }{1+log_{2}\left(cos\theta +1\right)}$	Where θ is the turning angle between 2 steps vector	[44,73]

## Appendix D. Classification Algorithms Details

**Table A5:** Potential subsets of features obtained by different feature selection methods.

ID	Feature Name	All Features		RFE		f-classif		Chi2		Importance	
		**E**	**DR**	**E**	**DR**	**E**	**DR**	**E**	**DR**	**E**	**DR**
1	Total distance	+	+	+	+	+	+	+	+	+	+
2	turn30_60	+	+	-	-	+	-	-	-	-	-
3	turn60_90	+	+	-	-	-	-	-	-	+	-
4	turn90_120	+	+	-	-	-	-	-	-	-	+
5	turn120	+	+	-	+	-	+	-	-	-	-
6	area	+	+	+	+	+	-	+	+	-	-
7	IU	+	+	-	-	-	-	+	-	-	-
8	ST	+	+	-	-	-	-	-	-	-	-
9	MSD	+	+	-	-	+	-	-	-	-	-
10	SI	+	+	-	-	-	-	-	-	+	-
11	FD	+	+	+	+	+	-	+	-	-	+
12	Average Speed	+	+	+	+	+	+	+	+	+	+
13	Speed Median	+	+	+	-	+	-	-	-	-	-
14	Speed Variance	+	+	-	-	-	+	-	+	+	+
15	Speed stdev	+	+	-	-	-	+	-	+	+	+
16	Max speed	+	+	-	+	-	+	-	-	-	-
17	Number of points	+	+	+	+	+	+	+	+	-	+
18	$QP_{4}$	+	+	+	-	-	-	+	+	+	-
19	TFC	NA	+	NA	-	NA	-	NA	-	NA	-
20	DFC	NA	+	NA	-	NA	-	NA	-	NA	-
21	TL	NA	+	NA	-	NA	-	NA	-	NA	-
22	Pace	NA	+	NA	-	NA	-	NA	-	NA	-

Table A6 presents the Features-Selection methods weight results (only the RFE output is boolean).

Table A7 presents a comparison of the considered classification algorithms in terms of precision, recall, F1-score, and ROC (ROC score provides the area under the Receiver Operating Characteristic curve and is a common metric in ML.) score. Random Forest, combined with RFE-method selected features’ list, had the best performance with 83.3% precision, 80% recall, 81% F1-score, and 81.6% ROC score.

Table A8 presents the count for each feature appearance in the subsets selected by the different feature selection algorithms, providing an indication for the prevalence of the features in the classification.

**Table A6:** Features-Selection methods weight results. Selected features marked in bold.

Features	Recursive Feature Elimination (RFE)	Univariate f-Classif	Univariate Chi2	Feature- Importance
Total distance	**TRUE**	**10.328**	**0.464**	**0.117**
turn30_60	FALSE	6.946	0.356	0.045
turn60_90	FALSE	6.803	0.351	0.008
turn90_120	FALSE	4.353	0.250	**0.089**
turn120	**TRUE**	**7.983**	0.392	0.038
area	**TRUE**	4.863	**0.763**	0.019
SI	FALSE	5.274	0.290	0.030
MSD	FALSE	6.542	0.341	0.026
IU	FALSE	2.139	0.383	0.004
ST	FALSE	0.100	0.007	0.001
FD	**TRUE**	6.927	0.355	**0.135**
Average Speed	**TRUE**	**9.489**	**0.440**	**0.130**
Speed Median	FALSE	4.900	0.274	0.034
Speed Variance	FALSE	**8.338**	**0.404**	**0.068**
Speed stdev	FALSE	**8.338**	**0.404**	**0.054**
Max speed	**TRUE**	**7.583**	0.379	0.041
Number of points	**TRUE**	**10.974**	**0.478**	**0.051**
$QP_{4}$	FALSE	1.118	**1.111**	0.001
TFC	FALSE	0.783	0.125	0.000
DFC	FALSE	0.393	0.027	0.046
TL	FALSE	5.659	0.306	0.025
Pace	FALSE	1.309	0.245	0.000

**Table A7:** Random-Forest classification model prediction results using different features selections list.

Features-Selection Model	Precision	Recall	F1-Score	ROC Score
All features	77.78%	73.68%	75.68%	76.32%
Recursive Feature Elimination(RFE)	83.33%	78.95%	81.08%	81.58%
Univariate Correlation f-classif	82.35%	73.68%	77.77%	78.94%
Univariate Correlation Chi2	77.78%	73.68%	75.68%	76.32%
Importance	73.68%	73.68%	73.68%	73.68%

**Table A8:** Selected features’ prevalence based of Features-Selection results.

ID	Feature	Prevalence
1	Total distance	4
2	Average Speed	4
3	area	3
4	FD	3
5	Number of points	3
6	Quadrant 4 points	3
7	Speed Median	2
8	turn30_60	1
9	turn60_90	1
10	SI	1
11	MSD	1
12	IU	1
13	Speed Variance	1
14	Speed stdev	1

## Appendix E. Focus Group Discussion Analysis

Below we present results of our analysis of the focus group transcriptions, identifying some common emerging themes.

In the first part of the FGD, four videos of dogs were shown to the participants in the following order: Lichi (H-group), Dream (C-group), Sia (H-group), and Laila (C-group). They were requested to discuss which of the shown dogs belongs to which groups. All participants of the FGD correctly identified the dogs’ classification.

Here are some of examples of relevant quotes:
“/.../ Lichi and Sia show more nervous movements than the others. This is showing to me they are stressed. Also their ears are flattened, especially Lichi. Also his body posture was showing me he was not feeling comfortable.” (**P1**)
“/.../ Lichi was moving chak! chak! chak! [abrupt hand gestures] from one angle to the other without stopping to explore” (**P1**)
“/.../ the first one [Lichi] had quite erratic exploration and was sniffing a lot so maybe [they are hyperactive] /.../ the last one [Laila] I would say no because she was exploring very calmly and more systematically than the first one [Lichi].” (**P2**)
“/.../ he [Lichi] was zapping from a corner to a corner to a corner. And this could be seen like impulsive behavior. And compulisuve behavior is the impossibility for the dog to stop. It is interesting to put them in front of something new, and then you see the impulsivity and compulsivity and everything. And he [Lichi] was not afraid, he was not aggressive, he was just, I will say “over” happy.” (**P3**)
“/.../ I agree Lichi shows hyperactivity. But like in humans, in dogs ADHD is a spectrum, you have severe ADHD and the grey zone, where its rather normal. For a better diagnosis, it’s better to look at a dog for 1 h to see whether it is able to stop the impulsive behavior. That is why we always also have house information from owner. So yes, we need a lot more information to characterize everything. From just looking at this movement we do not have the whole picture, that’s for sure. Even the vet can’t do it precisely, so of course the Blyzer cannot do it either.” (**P4**)

The participants also expressed positive attitudes concerning the approach and its usefulness in clinical settings. Here are some example quotes:
“/.../ In behavior we like very much objective assessment. We use grades, we use scales...That why it’s a very good tool to confirm our decision making. I do not see it replacing us in our practice.” (**P4**)
“/.../ It’s a great tool. But for me it’s not a tool to say the dog is hyperactive, but a tool that says that in this particular situation the dog is acting hyperactively. But I am searching for objective tools and in this context its really great start.” (**P1**)
“/.../ Because we are taking the same 3 min from all dogs, it is comparable. It will work, if we have lots of data. ” (**P4**)
“/.../ It’s a great tool to measure signs and symptoms objectively, and a small step towards the next level where we can make a diagnosis. It’s like you take a stethoscope, put it on the heart and you hear murmur, but that is not sufficient information to make a diagnosis. ” (**P2**)
“I think we all agree its a great tool, but [specifically] a great tool to measure signs or symptoms to go to the next level and to say it can make a diagnosis.” (**P4**)

Additional themes emerging from the discussion centered around:

- The potential of the approach for early detection:
  “/.../ you could also see this tool as a prevention…they can use your app on phone and like they film their dog quiet in the room when no-one is doing anything and maybe one day it will give them a score “your dog is hyper” if it has these symptoms and so they know it can happen /.../”
  (**P3**)
- The importance of further exploring the importance of social cues in a protocol for ADHD-like behavior testing.
  “/.../ The future protocols probably should not allow hand movement and petting the dog, to produce less social cues.”
  (**P2**)
  “/.../ for instance, for me I see in Lichie a dog who’s moving faster maybe because of the social cues that are there... It also could be the case that he is also socially impaired’, reacting to people. This should be taken into account.”
  (**P1**)
- The added value of the approach for communicating with owners:
  “/.../ I often talk to owners, explaining to them how the treatment will help. Having scores to show them would be good for the link with owners. So yes, it can be a great help for us...Of course, it’s not only hyperactivity that we should measure, but it is a good start.”
  (**P4**)
  I think it’s interesting and important for owners to see objective data on their dogs and I think its interesting to maybe in (chatters?) or general consultations, I am very interested in this for all these reasons and I also think about something else.
  (**P2**)

## References

[1] Polanczyk G., De Lima M.S., Horta B.L., Biederman J., Rohde L.A. (2007). The worldwide prevalence of ADHD: A systematic review and metaregression analysis. Am. J. Psychiatry.

[2] Faraone S.V., Sergeant J., Gillberg C., Biederman J. (2003). The worldwide prevalence of ADHD: Is it an American condition?. World Psychiatry.

[3] Colledge E., Blair R. (2001). The relationship in children between the inattention and impulsivity components of attention deficit and hyperactivity disorder and psychopathic tendencies. Personal. Individ. Differ..

[4] Saldana L., Neuringer A. (1998). Is instrumental variability abnormally high in children exhibiting ADHD and aggressive behavior?. Behav. Brain Res..

[5] Willcutt E.G., Carlson C.L. (2005). The diagnostic validity of attention-deficit/hyperactivity disorder. Clin. Neurosci. Res..

[6] Barkley R.A. (2003). Issues in the diagnosis of attention-deficit/hyperactivity disorder in children. Brain Dev..

[7] Edwards M.C., Gardner E.S., Chelonis J.J., Schulz E.G., Flake R.A., Diaz P.F. (2007). Estimates of the validity and utility of the Conners’ Continuous Performance Test in the assessment of inattentive and/or hyperactive-impulsive behaviors in children. J. Abnorm. Child Psychol..

[8] Van Der Ende J., Verhulst F.C. (2005). Informant, gender and age differences in ratings of adolescent problem behaviour. Eur. Child Adolesc. Psychiatry.

[9] Emser T.S., Johnston B.A., Steele J.D., Kooij S., Thorell L., Christiansen H. (2018). Assessing ADHD symptoms in children and adults: Evaluating the role of objective measures. Behav. Brain Funct..

[10] Sempere-Tortosa M., Fernández-Carrasco F., Mora-Lizán F., Rizo-Maestre C. (2020). Objective Analysis of Movement in Subjects with ADHD. Multidisciplinary Control Tool for Students in the Classroom. Int. J. Environ. Res. Public Health.

[11] Hoppe N., Bininda-Emonds O., Gansloßer U. (2017). Correlates of attention deficit hyperactivity disorder (ADHD)-like behavior in domestic dogs: First results from a questionnaire-based study. Vet. Med. Open J..

[12] Vas J., Topál J., Péch E., Miklósi A. (2007). Measuring attention deficit and activity in dogs: A new application and validation of a human ADHD questionnaire. Appl. Anim. Behav. Sci..

[13] Puurunen J., Sulkama S., Tiira K., Araujo C., Lehtonen M., Hanhineva K., Lohi H. (2016). A non-targeted metabolite profiling pilot study suggests that tryptophan and lipid metabolisms are linked with ADHD-like behaviours in dogs. Behav. Brain Funct..

[14] Dinwoodie I.R., Dwyer B., Zottola V., Gleason D., Dodman N.H. (2019). Demographics and comorbidity of behavior problems in dogs. J. Vet. Behav..

[15] Luescher U.A. (1993). Hyperkinesis in dogs: Six case reports. Can. Vet. J..

[16] Landsberg G.M., Hunthausen W. (1997). Handbook of Behaviour Problems of the Dog and Cat.

[17] Overall K. (2013). Manual of Clinical Behavioral Medicine for Dogs and Cats-E-Book.

[18] Wright H.F., Mills D.S., Pollux P.M. (2011). Development and Validation of a Psychometric Tool for Assessing Impulsivity in the Domestic Dog (*Canis familiaris*). Int. J. Comp. Psychol..

[19] Pageat P. (1998). Pathologie du comportement du chien.

[20] Bamberger M., Houpt K.A. (2006). Signalment factors, comorbidity, and trends in behavior diagnoses in dogs: 1644 cases (1991–2001). J. Am. Vet. Med. Assoc..

[21] Khoshnegah J., Azizzadeh M., Gharaie A.M. (2011). Risk factors for the development of behavior problems in a population of Iranian domestic dogs: Results of a pilot survey. Appl. Anim. Behav. Sci..

[22] New J.C., Salman M., King M., Scarlett J.M., Kass P.H., Hutchison J.M. (2000). Characteristics of shelter-relinquished animals and their owners compared with animals and their owners in US pet-owning households. J. Appl. Anim. Welf. Sci..

[23] Patronek G.J., Glickman L.T., Beck A.M., McCabe G.P., Ecker C. (1996). Risk factors for relinquishment of dogs to an animal shelter. J. Am. Vet. Med. Assoc..

[24] Masson S., Gaultier E. (2018). Retrospecive Study on Hypersensitivity-Hyperactivity Syndrome in Dogs: Long-term Outcome of High Dose Fluoxetine treatment and Proposal of a Clinical Score. Dog Behav..

[25] Hsu Y., Serpell J.A. (2003). Development and validation of a questionnaire for measuring behavior and temperament traits in pet dogs. J. Am. Vet. Med. Assoc..

[26] Ley J.M., Bennett P.C., Coleman G.J. (2009). A refinement and validation of the Monash Canine Personality Questionnaire (MCPQ). Appl. Anim. Behav. Sci..

[27] Jones A. (2008). Development and Validation of a Dog Personality Questionnaire. Ph.D. Thesis.

[28] Lit L., Schweitzer J.B., Iosif A.M., Oberbauer A.M. (2010). Owner reports of attention, activity, and impulsivity in dogs: A replication study. Behav. Brain Funct..

[29] Tiihonen J., Rautiainen M., Ollila H., Repo-Tiihonen E., Virkkunen M., Palotie A., Pietiläinen O., Kristiansson K., Joukamaa M., Lauerma H. (2015). Genetic background of extreme violent behavior. Mol. Psychiatry.

[30] Peremans K., Audenaert K., Coopman F., Blanckaert P., Jacobs F., Otte A., Verschooten F., van Bree H., van Heeringen K., Mertens J. (2003). Estimates of regional cerebral blood flow and 5-HT2A receptor density in impulsive, aggressive dogs with 99m Tc-ECD and 123 I-5-I-R91150. Eur. J. Nucl. Med. Mol. Imaging.

[31] LaHoste G.J., Swanson J., Wigal S.B., Glabe C., Wigal T., King N., Kennedy J. (1996). Dopamine D4 receptor gene polymorphism is associated with attention deficit hyperactivity disorder. Mol. Psychiatry.

[32] Hejjas K., Vas J., Topál J., Szántai E., Rónai Z., Székely A., Kubinyi E., Horváth Z., Sasvari-Szekely M., Miklosi A. (2007). Association of polymorphisms in the dopamine D4 receptor gene and the activity-impulsivity endophenotype in dogs. Anim. Genet..

[33] Ito H., Nara H., Inoue-Murayama M., Shimada M.K., Koshimura A., Ueda Y., Kitagawa H., Takeuchi Y., Mori Y., Murayama Y. (2004). Allele frequency distribution of the canine dopamine receptor D4 gene exon III and I in 23 breeds. J. Vet. Med. Sci..

[34] Bunford N., Csibra B., Peták C., Ferdinandy B., Miklósi Á., Gácsi M. (2019). Associations among behavioral inhibition and owner-rated attention, hyperactivity/impulsivity, and personality in the domestic dog (*Canis familiaris*). J. Comp. Psychol..

[35] Mège C. (2003). Pathologie comportementale du chien.

[36] Zamansky A., Sinitca A.M., Kaplun D.I., Plazner M., Schork I.G., Young R.J., de Azevedo C.S. (2019). Analysis of dogs’ sleep patterns using convolutional neural networks. Proceedings of the International Conference on Artificial Neural Networks.

[37] Buchanan K., Burt de Perera T., Carere C., Carter T., Hailey A., Hubrecht R., Jennings D., Metcalfe N., Pitcher T., Peron F. (2012). Guidelines for the treatment of animals in behavioural research and teaching. Anim. Behav..

[38] Bleuer-Elsner S., Zamansky A., Fux A., Kaplun D., Romanov S., Sinitca A., Masson S., van der Linden D. (2019). Computational Analysis of Movement Patterns of Dogs with ADHD-Like Behavior. Animals.

[39] Zamansky A., Bleuer-Elsner S., Masson S., Amir S., Magen O., van der Linden D. (2018). Effects of anxiety on canine movement in dog-robot interactions. Anim. Behav. Cogn..

[40] Ren S., He K., Girshick R., Sun J. (2016). Faster r-cnn: Towards real-time object detection with region proposal networks. IEEE Trans. Pattern Anal. Mach. Intell..

[41] Howard A.G., Zhu M., Chen B., Kalenichenko D., Wang W., Weyand T., Andreetto M., Adam H. (2017). Mobilenets: Efficient convolutional neural networks for mobile vision applications. arXiv.

[42] Chandrashekar G., Sahin F. (2014). A survey on feature selection methods. Comput. Electr. Eng..

[43] Nathan R. (2008). An emerging movement ecology paradigm. Proc. Natl. Acad. Sci. USA.

[44] Almeida P.J., Vieira M.V., Kajin M., Forero-Medina G., Cerqueira R. (2010). Indices of movement behaviour: Conceptual background, effects of scale and location errors. Zoologia.

[45] Pavlyuk D. (2019). Feature selection and extraction in spatiotemporal traffic forecasting: A systematic literature review. Eur. Transp. Res. Rev..

[46] Jović A., Brkić K., Bogunović N. (2015). A review of feature selection methods with applications. Proceedings of the 2015 38th International Convention on Information and Communication Technology, Electronics and Microelectronics (MIPRO).

[47] Kohavi R., John G.H. (1997). Wrappers for feature subset selection. Artif. Intell..

[48] Kotsiantis S.B., Zaharakis I., Pintelas P. (2007). Supervised machine learning: A review of classification techniques. Emerg. Artif. Intell. Appl. Comput. Eng..

[49] Wong S.F., Cipolla R. (2007). Extracting spatiotemporal interest points using global information. Proceedings of the 2007 IEEE 11th International Conference on Computer Vision.

[50] Naghibi S.A., Pourghasemi H.R., Dixon B. (2016). GIS-based groundwater potential mapping using boosted regression tree, classification and regression tree, and random forest machine learning models in Iran. Environ. Monit. Assess..

[51] Kamberelis G., Dimitriadis G. (2013). Focus Groups: From Structured Interviews to Collective Conversations.

[52] Rosenbaum S., Cockton G., Coyne K., Muller M., Rauch T. Focus groups in HCI: Wealth of information or waste of resources?. Proceedings of the CHI’02 Extended Abstracts on Human Factors in Computing Systems.

[53] Guest G., Namey E., McKenna K. (2017). How many focus groups are enough? Building an evidence base for nonprobability sample sizes. Field Methods.

[54] Kaplun D., Sinitca A., Zamansky A., Bleuer-Elsner S., Plazner M., Fux A., van der Linden D. Animal health informatics: Towards a generic framework for automatic behavior analysis. Proceedings of the 12th International Conference on Health Informatics (HEALTHINF 2019).

[55] Shemesh Y., Sztainberg Y., Forkosh O., Shlapobersky T., Chen A., Schneidman E. (2013). High-order social interactions in groups of mice. Elife.

[56] Mealin S., Domínguez I.X., Roberts D.L. (2016). Semi-supervised classification of static canine postures using the Microsoft Kinect. Proceedings of the Third International Conference on Animal-Computer Interaction.

[57] Barnard S., Calderara S., Pistocchi S., Cucchiara R., Podaliri-Vulpiani M., Messori S., Ferri N. (2016). Quick, accurate, smart: 3D computer vision technology helps assessing confined animals’ behaviour. PLoS ONE.

[58] Karl S., Boch M., Zamansky A., van der Linden D., Wagner I.C., Völter C.J., Lamm C., Huber L. (2020). Exploring the dog-human relationship by combining fMRI, eye-tracking and behavioural measures. Sci. Rep..

[59] Leaver S., Reimchen T. (2008). Behavioural responses of Canis familiaris to different tail lengths of a remotely-controlled life-size dog replica. Behaviour.

[60] Gergely A., Petró E., Topál J., Miklósi Á. (2013). What are you or who are you? The emergence of social interaction between dog and an unidentified moving object (UMO). PLoS ONE.

[61] Kubinyi E., Miklósi Á., Kaplan F., Gácsi M., Topál J., Csányi V. (2004). Social behaviour of dogs encountering AIBO, an animal-like robot in a neutral and in a feeding situation. Behav. Process..

[62] Chen T., Shi X., Wong Y.D. (2019). Key feature selection and risk prediction for lane-changing behaviors based on vehicles’ trajectory data. Accid. Anal. Prev..

[63] Lee Y., Lim W. (2017). Shoelace Formula: Connecting the Area of a Polygon and the Vector Cross Product. Math. Teach..

[64] Guiñón J.L., Ortega E., García-Antón J., Pérez-Herranz V. (2007). Moving average and Savitzki-Golay smoothing filters using Mathcad. Pap. ICEE.

[65] Saalfeld A. (1999). Topologically consistent line simplification with the Douglas-Peucker algorithm. Cartogr. Geogr. Inf. Sci..

[66] Loretto D., Vieira M.V. (2005). The effects of reproductive and climatic seasons on movements in the black-eared opossum (Didelphis aurita Wied-Neuwied, 1826). J. Mammal..

[67] Batschelet E. (1981). Circular Statistics in Biology.

[68] Bovet P., Benhamou S. (1988). Spatial analysis of animals’ movements using a correlated random walk model. J. Theor. Biol..

[69] Slade N.A., Swihart R.K. (1983). Home range indices for the hispid cotton rat (*Sigmodon hispidus*) in northeastern Kansas. J. Mammal..

[70] Tremblay Y., Roberts A.J., Costa D.P. (2007). Fractal landscape method: An alternative approach to measuring area-restricted searching behavior. J. Exp. Biol..

[71] Labatut V. (2018). Continuous average Straightness in spatial graphs. J. Complex Netw..

[72] Benhamou S. (2004). How to reliably estimate the tortuosity of an animal’s path: Straightness, sinuosity, or fractal dimension?. J. Theor. Biol..

[73] Nams V.O. (1996). The VFractal: A new estimator for fractal dimension of animal movement paths. Landsc. Ecol..

